# Prevalence and associated factors for burnout among attending general surgeons: a national cross-sectional survey

**DOI:** 10.1186/s12913-020-06024-5

**Published:** 2021-01-07

**Authors:** Suleyman Utku Celik, Alperen Aslan, Eylul Coskun, Beyza Nur Coban, Zeynep Haner, Selin Kart, Mahmoud N. I. Skaik, Merve Didem Kocer, Bahar Busra Ozkan, Cihangir Akyol

**Affiliations:** 1 Department of General Surgery, Ankara University School of Medicine, Ibn-i Sina Hospital, 06100 Ankara, Turkey; 2 Department of General Surgery, Gulhane Training and Research Hospital, Ankara, Turkey; 3 Ankara University School of Medicine, Ankara, Turkey

**Keywords:** Burnout, General surgery, Maslach burnout inventory, Work-life balance

## Abstract

**Background:**

Burnout resulting from long-term and unmanaged workplace stress is high among healthcare professionals, especially surgeons, and affects both individuals and the quality of patient care. The objective of this study was to determine the prevalence and associated factors for burnout among attending general surgeons and to identify possible preventive strategies.

**Methods:**

A national cross-sectional survey using a 35-item questionnaire was conducted among members of the Turkish Surgical Society. The survey evaluated demographics, professional and practice characteristics, social participation, and burnout as well as interventions to deal with burnout. Burnout was defined as a high score on the emotional exhaustion (EE) and/or depersonalization (DP) subscales. Surgeons with high scores on both the EE and DP and a low score on personal accomplishment (PA) were considered to have severe burnout.

**Results:**

Six hundred fifteen general surgeons completed the survey. The median EE, DP, and PA scores were 34 (IQR, 20–43), 9 (IQR, 4–16), and 36 (IQR, 30–42), respectively. Overall, the prevalence of burnout and severe burnout were 69.1 and 22.0%, respectively. On multivariable analysis, factors independently associated with burnout were working in a training and research hospital (OR = 3.34; *P* < 0.001) or state hospital (OR = 2.77; *P* = 0.001), working ≥ 60 h per week (OR = 1.57; *P* = 0.046), and less frequent participation in social activities (OR = 3.65; *P* < 0.001).

**Conclusions:**

Burnout is an important problem among general surgeons with impacts and consequences for professionals, patients, and society. Considering that burnout is a preventable condition, systematic efforts to identify at-risk populations and to develop strategies to address burnout in surgeons are needed.

**Supplementary Information:**

The online version contains supplementary material available at 10.1186/s12913-020-06024-5.

## Background

Burnout is an occupational condition resulting from long-term and unmanaged work-related stress and is characterized by exhaustion of physical/emotional strength, feelings of cynicism and disengagement from work, or reduced professional efficacy [1, 2]. Healthcare professionals are especially at high risk for burnout due to high patient volume, working long hours, chronic exposure to human suffering, and facing life or death situations on a regular basis as well as the lack of work-life balance and poor social support [3–5]. Beyond its negative effect on the mental health and well-being of physicians, burnout can also contribute to reduced physician performance and quality of life, failure of quality of interactions with patients and other members of the healthcare team, and increased medical errors, which ultimately affects the quality of care and patient-related outcomes [1, 2, 6–8]. In addition, burnout is associated with increased rates of depression, abuse of alcohol and other substances, and risk for suicidal behaviors [1, 9]. Existing data also suggest that this condition may contribute to early retirement [6, 10].

Burnout is particularly common in surgical specialties [11]. Numerous studies of burnout among nearly all surgical specialties have been conducted, and these studies reported a wide range of burnout prevalence. In a national study of 7905 surgeons across all subspecialties, the authors found a rate of burnout of approximately 40% among American surgeons (higher risk in trauma surgeons, urologists, otolaryngologists, vascular surgeons, and general surgeons) [6]. Recently, awareness of burnout has increased globally, and the knowledge and ability to recognize physicians suffering from this occupational phenomenon have improved [2, 11].

The majority of evidence on burnout in the field of surgery is particularly based on surgery residents and trainees, who also struggle to improve their medical and academic knowledge during their training [6, 11–13]. In addition, surgical specialties, which have their own characteristics, such as cultures, working processes, and demands, have been mostly evaluated collectively [12, 14]. However, almost no study has focused solely on attendings and more experienced professionals working in general surgery. To address this, we conducted a national cross-sectional study of burnout specifically among attending general surgeons and assessed the prevalence of burnout. In addition, we evaluated the personal and professional characteristics associated with burnout and aimed to identify possible preventive strategies.

## Methods

### Individuals

In April 2019, all members of the Turkish Surgical Society were invited to participate in an online, self-administered survey on occupational burnout (http://surveymonkey.com). Participation was optional and anonymous. Residents were not included in the study. Subjects who failed to complete the questionnaire were excluded from the study.

### Questionnaire

A 35-item questionnaire was developed to collect data about demographics, professional and practice characteristics, social participation, and burnout.

The first part of the questionnaire consisted of 13 items that assessed demographic factors, such as age, sex, marital and children status; practice characteristics, including academic title, workplace, work hours per week [3, 15], and sleep duration [16]; and leisure activities, such as hobbies and socializing. The last question, which is an optional, open-ended, and free response, was about suggestions on how to combat burnout (Supplementary file 1).

In the second part, burnout was assessed using the validated Turkish version of the 22-item Maslach Burnout Inventory-Human Services Survey (MBI-HSS) for Medical Personnel (MP). The MBI-HSS for MP was adapted for use in medical settings by using the original questions with only slightly modified wording [17]. It has validity evidence for use in health-related occupations and has been used in many studies on burnout in nearly all surgical disciplines. The license for this tool was purchased from Mind Garden (http://www.mindgarden.com). The MBI-HSS assesses three core aspects of the burnout syndrome: emotional exhaustion (EE) (9 items), depersonalization (DP) (5 items), and lack of personal accomplishment (PA) (8 items). Each dimension of burnout is measured by several items, and all items are rated on a 7-point Likert scale ranging from 0 (never) to 6 (every day). In addition to continuous subscale measures, ranging from 0 to 54 for EE, 0 to 30 for DP, and 0 to 48 for PA, we used previously described cutoffs for each dimension of burnout to define them as low, moderate, and high (i.e., for EE, ≤ 18 = low, 19–26 = moderate, and ≥ 27 = high; for DP, ≤ 5 = low, 6–9 = moderate, and ≥ 10 = high; and for PA, ≤ 33 = low, 34–39 = moderate, and ≥ 40 = high) [7, 18]. Scores in the highest tertile for both EE and DP and in the lowest tertile for PA indicate greater risk for occupational burnout.

There is wide variability in the definition of burnout [18]. In the present study, three burnout components were calculated and interpreted separately. As a reduced PA score is descriptive of, but does not predict, professional burnout, burnout was defined as a high score on the EE and/or DP subscales [6, 10, 15, 17, 19]. In addition, we defined “severe burnout” as a subset of burnout, which represented high scores on both the EE and DP subscales and a low score on the PA subscale.

The reliability and validity of burnout inventory has been demonstrated in prior studies [17, 20]. The questionnaire was also pilot-tested on a convenience sample of five attending general surgeons from the researchers’ institute to ensure the clarity and coherence of its items.

### Data collection

A description of this national cross-sectional study and web-link to the survey were advertised on the Turkish Surgical Society’s website (https://www.turkcer.org.tr). Responses were collected anonymously from April 1 to April 30, 2019. In addition to recurring emails, reminders were sent to participants at regular intervals if they did not complete the survey within the allocated time. A maximum of two reminders were sent to nonresponders 7 and 14 days after the initial invitation. After 30 days, the survey was set offline and the data were locked. No incentive was offered for survey participation.

### Ethical approval

This study was approved by the Ankara University School of Medicine Undergraduate Student Research Ethics Committee (approval number: 72189195–050.03.04-E.5754). Completion of the survey was deemed consent by the participants to be involved in the study.

### Statistical analysis

Data were extracted from the online database and imported into the Statistical Package for the Social Sciences, version 16.0 (IBM^®^, Chicago, USA) for analysis. Figures were created using GraphPad Prism, version 8.0 (GraphPad Software Inc., California, USA).

Descriptive statistics were presented as the means ± standard deviations (SDs), medians and interquartile ranges (IQRs), and frequencies (%). Examinations of normal distribution assumptions for continuous variables were visually assessed with quantile-quantile plots and histograms and confirmed with the Shapiro-Wilk test. Associations between variables were evaluated using the Mann-Whitney U and Kruskal-Wallis tests (for continuous variables) or Pearson χ^2^ and Fisher exact tests (for categorical variables), where appropriate.

The association of burnout and severe burnout with demographics, professional and practice characteristics, and social participation were analyzed with logistic regression analysis. First, each covariate was analyzed in a univariate model, and all variables with *P* value < 0.20 were included in the final multivariate logistic regression model to determine independent factors. Odds ratios (ORs) of statistically significant predictors were presented with 95% confidence intervals (CIs). All tests were two-sided, and a *P* value < 0.05 was considered statistically significant.

## Results

Of the 3815 Turkish Surgical Society members invited, a total of 615 actively practicing attending general surgeons (16.1%) completed the survey. Table 1 presents the sociodemographic and occupational characteristics of the sample. The mean age of the respondents was 45.3 ± 8.9 years (range, 27–74 years), and the vast majority were male (*n* = 543, 88.3%). In all, 92.0% (*n* = 566) of the respondents were married or had a partner, and 86.0% (*n* = 529) had at least one child. With regard to practice characteristics, 27.8% of the surgeons worked in a training and research hospital (TRH), 20.2% worked in a state hospital, 25.2% worked in a university hospital, and 26.8% worked in a private hospital. Most of the surgeons (62.0%) were specialists, 136 (22.6%) were assistant or associate professors, and 95 (15.4%) were full professors.

**Table 1:** Sociodemographic and occupational characteristics of the general surgeons

Characteristics	
Age (years), mean ± SD	45.3 ± 8.9 (range, 27–74)
Sex, n (%)	
Female	72 (11.7)
Male	543 (88.3)
Marital status, n (%)	
Single	49 (8.0)
Married (or partnered)	566 (92.0)
Children, n (%)	
No	86 (14.0)
Yes	529 (86.0)
Academic title, n (%)	
Specialist	381 (62.0)
Assistant/Associate professor	139 (22.6)
Professor	95 (15.4)
Workplace, n (%)	
Training and research hospital	171 (27.8)
State hospital	124 (20.2)
University hospital	155 (25.2)
Private hospital	165 (26.8)
Work hours per week, n (%)	
< 60	392 (63.7)
≥ 60	223 (36.3)
Sleep duration (hours), mean ± SD	6.4 ± 0.9 (range, 3.5–10.0)
Comorbidity, n (%)	
No	416 (67.6)
Yes	199 (32.4)
Smoking status, n (%)	
No	428 (69.6)
Yes	187 (30.4)
Do you have a specific hobby outside of work?, n (%)	
No	292 (47.5)
Yes	323 (52.5)
Do you participate in any social activities outside of work (at least once a week)?, n (%)	
No	317 (51.5)
Yes	298 (48.5)

**Fig. 1:**
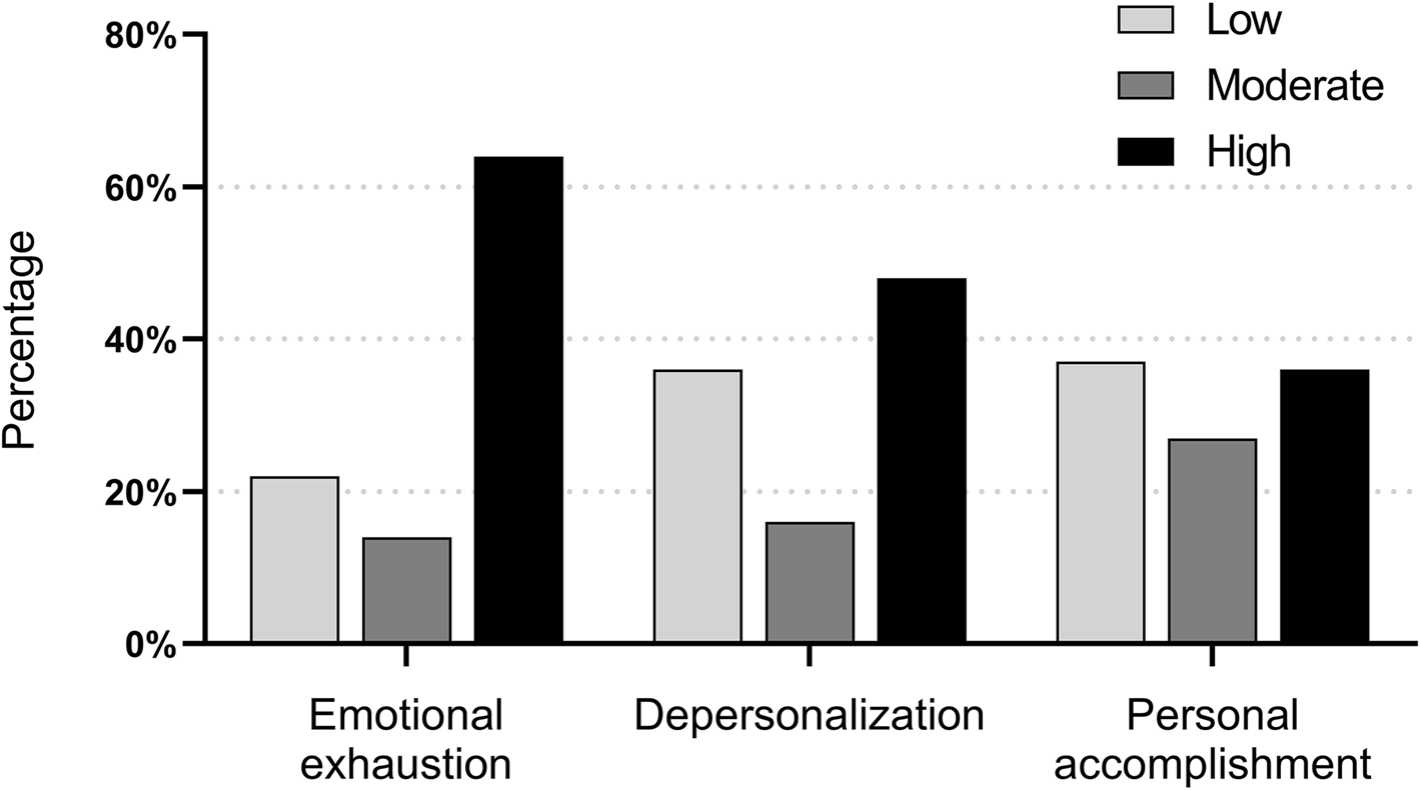
MBI-HSS results showing the emotional exhaustion, depersonalization, and personal accomplishment subscales of attending general surgeons categorized into low, moderate, and high

Table 2 and Fig. 1 describe the distribution of low, moderate, and high scores for the 3 burnout constructs of the MBI-HSS among the attending general surgeons. The median EE, DP, and PA scores were 34 (IQR, 20–43), 9 (IQR, 4–16), and 36 (IQR, 30–42), respectively. In addition, 63.9% (*n* = 393) of the respondents had high scores for EE, 47.8% (*n* = 294) had high scores for DP, and 36.9% (*n* = 227) exhibited low scores for PA.

**Table 2:** Maslach Burnout Inventory-Human Services Survey subscale scores among the sample of Turkish general surgeons

Subscale	Low	Moderate	High	Mean ± SD Median (IQR)
Emotional exhaustion	138 (22.4%)	84 (13.7%)	393 (63.9%)	31.4 ± 14.4 34 (20–43)
Depersonalization	224 (36.4%)	97 (15.8%)	294 (47.8%)	10.2 ± 7.6 9 (4–16)
Personal accomplishment	227 (36.9%)	169 (27.5%)	219 (35.6%)	35.6 ± 8.0 36 (30**–**42)

*SD* standard deviation, *IQR* interquartile range

Table 3 shows the median score for each burnout domain according to sociodemographic and occupational characteristics. Being < 40 years of age, being childless, being a specialist, working in a TRH or state hospital, working ≥ 60 h per week, sleeping < 7 h per night, not having a specific hobby, and not participating in a social activity outside of work were significantly associated with higher levels of EE and DP. Additionally, all but two of the abovementioned characteristics (work hours and sleep duration) were significantly associated with a lower level of PA.

**Table 3:** Sociodemographic and occupational characteristics associated with burnout subscales among general surgeons

Surgeon characteristics	Emotional exhaustion		Depersonalization		Personal accomplishment	
	Median (IQR)	***P***	Median (IQR)	***P***	Median (IQR)	***P***
Age, years		**0.010**		**< 0.001**		**< 0.001**
< 40	36 (24–42)		12 (6–17)		34 (29–40)	
≥ 40	31 (18–44)		7 (3–15)		38 (31–43)	
Sex		0.273		0.709		0.487
Female	35 (26–42)		8.5 (4–15)		38 (32–42)	
Male	33 (19–44)		9 (3–16)		36 (30–42)	
Marital status		0.543		0.927		0.110
Single	36 (21–42)		8 (4–15)		36 (30–38)	
Married (or partnered)	33 (20–43)		9 (3–16)		36 (30–42)	
Children		**0.003**		**0.023**		**0.022**
No	38 (28–44)		11 (5–18)		35 (30–39)	
Yes	32 (19–43)		8 (3–16)		37 (30–42)	
Academic title		**< 0.001**		**< 0.001**		**< 0.001**
Specialist	37 (23–45)		11 (5–18)		35 (30–41)	
Assistant/Associate professor	31 (19–40)		7 (3–14)		38 (31–42)	
Professor	24 (12–36)		4 (1–10)		40 (32–45)	
Workplace		**< 0.001**		**< 0.001**		**< 0.001**
TRH	37 (29–46)		13 (6–20)		33 (28–38)	
State hospital	39 (24–46)		13 (6–18)		35 (29–42)	
University hospital	31 (19–42)		7 (3–12)		37 (31–42)	
Private hospital	25 (13–37)		5 (2–11)		40 (34–43)	
Work hours per week, hours		**0.001**		**0.028**		0.785
< 60	31 (18–42)		8 (3–16)		36 (30–42)	
≥ 60	37 (24–45)		10 (4–17)		37 (30–42)	
Sleep duration, hours		**< 0.001**		**0.003**		0.093
< 7	36 (23–45)		10 (4–17)		36 (30–42)	
≥ 7	30 (16–41)		7 (3–14)		37 (31–42)	
Comorbidity		0.925		0.848		0.072
No	34 (20–43)		9 (4–16)		36 (30–42)	
Yes	33 (21–44)		9 (3–16)		38 (30–43)	
Smoking status		**0.006**		**0.008**		**0.043**
No	31 (19–42)		8 (3–16)		37 (30–43)	
Yes	36 (23–45)		10 (4–17)		36 (30–40)	
Specific hobby		**0.003**		**0.031**		**< 0.001**
No	36 (24–45)		10 (4–17)		35 (29–41)	
Yes	31 (17–42)		8 (3–15)		38 (32–43)	
Social activity		**< 0.001**		**< 0.001**		**< 0.001**
No	38 (28–45)		11 (5–18)		34 (28–39)	
Yes	26 (14**–**38)		6 (2**–**13)		39 (34**–**44)	

*IQR* interquartile range, *TRH* training and research hospital

**Fig. 2:**
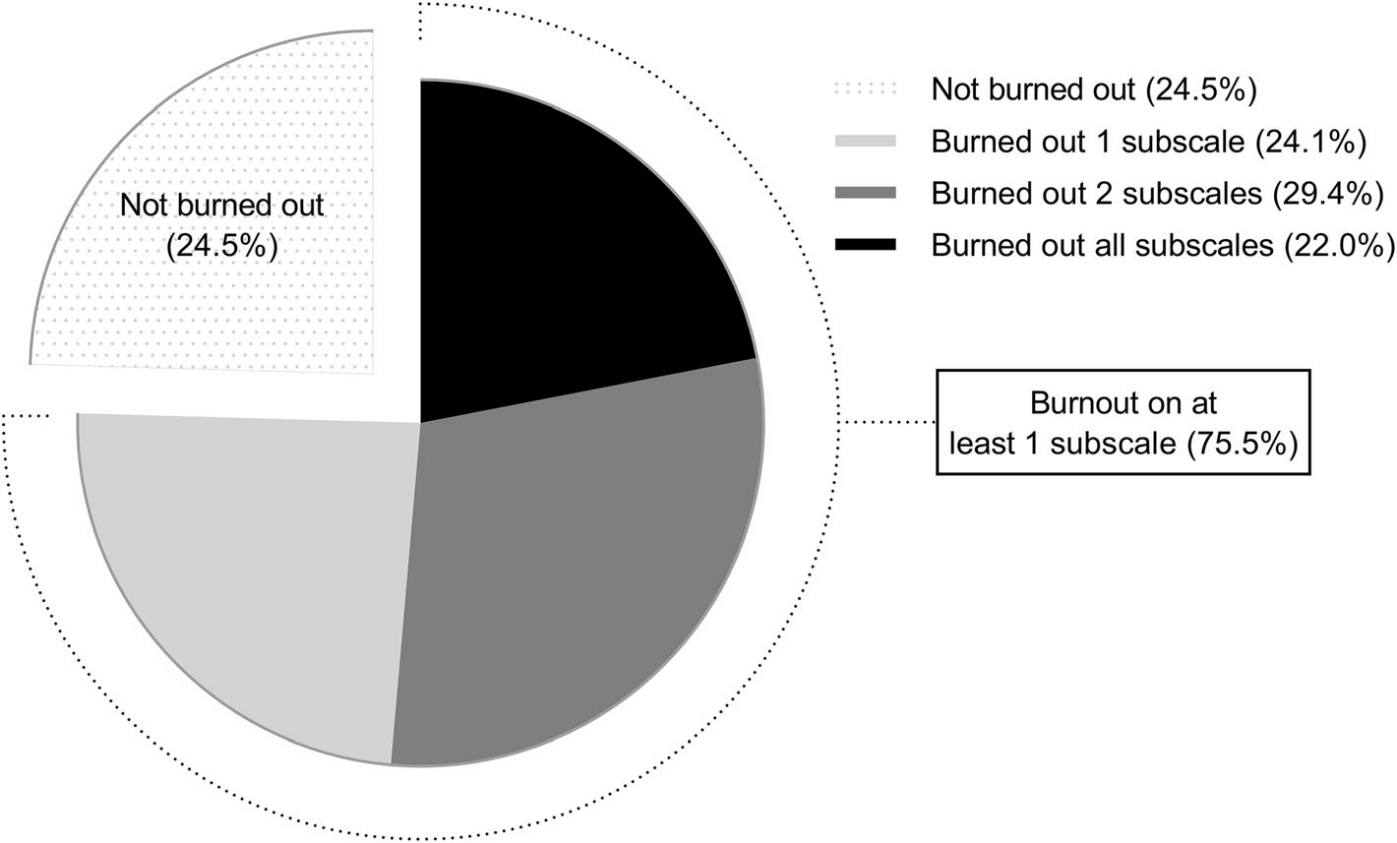
Percentage of attending general surgeons with severe burnout (high scores on both the emotional exhaustion and depersonalization subscales and a low score on the personal accomplishment subscale) (black) and those of surgeons who are burned out on 0, 1, or 2 of the subscales

The prevalence of burnout among attending general surgeons was 69.1% (95% CI, 65.3–72.6%) and the prevalence of severe burnout was 22.0% (95% CI, 18.8–25.3%). Among our survey participants, 75.5% had a high score in the EE or DP dimension or a low score in the PA dimension. In contrast, 24.5% of the respondents had low scores in the EE and DP subscales, and a high score in the PA subscale (Fig. 2).

Attending general surgeons younger than 40 years of age were more likely to experience burnout than older counterparts (79.4% vs. 65.2%; *P* = 0.001). Female gender (*P* = 0.012), being childless (*P* = 0.004), academic title (*P* < 0.001), workplace (*P* < 0.001), working ≥ 60 h per week (*P* < 0.001), sleeping < 7 h per night (*P* = 0.003), not having a specific hobby (*P* = 0.005), and not participating in a social activity (*P* < 0.001) were significantly associated with burnout. There were no significant differences in burnout rates by marital status, comorbidity, or smoking status. Attending general surgeons with severe burnout were significantly younger in age (*P* < 0.001) and more married or partnered (*P* = 0.038) compared with those who were not burned out. Higher levels of severe burnout were observed in specialists (25.7%) and assistant or associate professors (20.1%) compared to full professors (9.5%) (*P* = 0.002). Those who worked in a TRH (34.5%) or state hospital (27.4%) were more likely to exhibit severe burnout compared to those who worked in a university hospital (16.1%) or a private hospital (10.3%, *P* < 0.001). Attending general surgeons having a specific hobby (16.7% vs. 27.7%; *P* < 0.001) and a social life (12.1% vs. 31.2%; *P* < 0.001) had significantly lower levels of severe burnout (Table 4).

**Table 4:** Sociodemographic and occupational characteristics associated with burnout and severe burnout

Surgeon characteristics	Burnout (*n* = 425, 69.1%)^†^		Severe burnout (*n* = 135, 22.0%)^††^	
	n (%)	***P***	n (%)	***P***
Age, years		**0.001**		**< 0.001**
< 40	135 (79.4%)		57 (33.5%)	
≥ 40	290 (65.2%)		78 (17.5%)	
Sex		**0.012**		0.079
Female	59 (81.9%)		10 (13.9%)	
Male	366 (67.4%)		125 (23.0%)	
Marital status		0.182		**0.038**
Single	38 (77.6%)		5 (10.2%)	
Married (or partnered)	387 (68.4%)		130 (23.0%)	
Children		**0.004**		0.753
No	71 (82.6%)		20 (23.3%)	
Yes	354 (66.9%)		115 (21.7%)	
Academic title		**< 0.001**		**0.002**
Specialist	286 (75.1%)		98 (25.7%)	
Assistant/Associate professor	93 (66.9%)		28 (20.1%)	
Professor	46 (48.4%)		9 (9.5%)	
Workplace		**< 0.001**		**< 0.001**
TRH	141 (82.5%)		59 (34.5%)	
State hospital	100 (80.6%)		34 (27.4%)	
University hospital	102 (65.8%)		25 (16.1%)	
Private hospital	82 (49.7%)		17 (10.3%)	
Work hours per week		**< 0.001**		0.832
< 60	251 (64.0%)		85 (21.7%)	
≥ 60	174 (78.0%)		50 (22.4%)	
Sleep duration, hours		**0.003**		0.061
< 7	261 (73.9%)		87 (24.6%)	
≥ 7	164 (62.6%)		48 (18.3%)	
Comorbidity		0.565		0.276
No	299 (68.4%)		101 (23.1%)	
Yes	126 (70.8%)		34 (19.1%)	
Smoking status		0.140		0.403
No	288 (67.3%)		90 (21.0%)	
Yes	137 (73.3%)		45 (24.1%)	
Specific hobby		**0.005**		**< 0.001**
No	218 (74.7%)		81 (27.7%)	
Yes	207 (64.1%)		54 (16.7%)	
Social activity		**< 0.001**		**< 0.001**
No	264 (83.3%)		99 (31.2%)	
Yes	161 (54.0%)		36 (12.1%)	

*TRH* training and research hospital

^†^ Burnout was defined as a high score on the emotional exhaustion and/or depersonalization subscales

^††^ Severe burnout was defined as high scores on the emotional exhaustion and depersonalization and a low score on personal accomplishment

Consequently, we performed a multivariate analysis to identify factors independently associated with burnout and severe burnout. Working in a TRH (OR = 3.34; 95% CI, 1.93–5.77, *P* < 0.001) or a state hospital (OR = 2.77; 95% CI, 1.53–5.04, *P* = 0.001) were associated with an increased risk for burnout. Surgeons working in a private practice were found to be at lower risk for burnout compared to those working in a TRH, state hospital, or university hospital. Surgeons working ≥ 60 h per week had 1.5 times higher odds of exhibiting burnout than those working < 60 h per week (OR = 1.57; 95% CI, 1.01–2.45, *P* = 0.046). Not participating in a social activity at least once per week increased the likelihood of burnout by 3.6 times (OR = 3.65; 95% CI, 2.39–5.58, *P* < 0.001). Factors associated with an increased risk for severe burnout included decreasing age (OR = 0.95; 95% CI, 0.92–0.98, *P* = 0.002), working in a TRH (OR = 3.56; 95% CI, 1.89–6.69, *P* < 0.001) or a state hospital (OR = 2.46; 95% CI, 1.24–4.86, *P* = 0.009), and not participating in a regular social activity (OR = 2.50; 95% CI, 1.55–4.02, *P* < 0.001) (Table 5).

**Table 5:** Multivariate logistic regression analysis of factors associated with burnout and severe burnout

Variable	Burnout^a^		Severe burnout^b^	
	Adjusted odds ratio (95% CI)	***P***	Adjusted odds ratio (95% CI)	***P***
Age	0.98 (0.95–1.01)	0.202	0.95 (0.92–0.98)	**0.002**
Sex				
Female	Reference		Reference	
Male	0.54 (0.26–1.10)	0.091	2.09 (0.95–4.59)	0.064
Marital status				
Single	–	–	Reference	
Married (or partnered)	–	–	2.25 (0.80–6.37)	0.124
Children				
No	Reference		–	–
Yes	0.83 (0.41–1.67)	0.833	–	–
Academic title				
Specialist	1.66 (0.86–3.20)	0.130	1.11 (0.44–2.75)	0.821
Assistant/Associate professor	1.31 (0.68–2.49)	0.408	1.09 (0.44–2.67)	0.852
Professor	Reference		Reference	
Workplace				
TRH	3.34 (1.93–5.77)	**< 0.001**	3.56 (1.89–6.69)	**< 0.001**
State hospital	2.77 (1.53–5.04)	**0.001**	2.46 (1.24–4.86)	**0.009**
University hospital	1.66 (0.93–2.95)	0.084	1.32 (0.62–2.78)	0.466
Private hospital	Reference		Reference	
Work hours per week				
< 60	Reference		–	–
≥ 60	1.57 (1.01–2.45)	**0.046**	–	–
Sleep duration	0.83 (0.66–1.03)	0.102	0.91 (0.72–1.17)	0.490
Specific hobby				
No	0.98 (0.64–1.49)	0.929	1.34 (0.85–2.11)	0.195
Yes	Reference		Reference	
Social activity				
No	3.65 (2.39–5.58)	**< 0.001**	2.50 (1.55–4.02)	**< 0.001**
Yes	Reference		Reference	

*TRH* training and research hospital

^a^ Burnout was defined as a high score on the emotional exhaustion and/or depersonalization subscales

^b^ Severe burnout was defined as high scores on the emotional exhaustion and depersonalization and a low score on personal accomplishment

Table 6 presents surgeons’ **s**uggestions for addressing occupational burnout. Thus, the main suggested strategies to prevent healthcare professionals from burnout were the development of a fair salary policy (84.2%), prevention of violence (78.0%), reforms for reducing medical malpractice lawsuits (75.2%), improvement of working conditions (64.2%), and reduction of patient volume (62.7%).

**Table 6:** Participants’ **s**uggestions for reducing occupational burnout

Suggestions	n (%)
Development of a fair salary policy	518 (84.2)
Prevention of violence against health care workers	480 (78.0)
Reforms for reducing medical malpractice lawsuits	463 (75.2)
Improvement of working conditions	395 (64.2)
Reduction of patient burden/volume	386 (62.7)
Provision of a sufficient number of medical staff	351 (57.3)
Prevention of workplace bullying (mobbing)	319 (51.8)
Regulation of working hours (regular working hours)	290 (47.1)
Communication skills training for health care workers	188 (30.5)
Regular psychiatric support for health care workers	124 (20.1)

## Discussion

The possible negative impact of burnout on the quality of care requires that every medical specialty that has its own professional characteristics be evaluated separately for burnout. This national cross-sectional study serves to comprehensively assess the prevalence and risk factors for burnout in the field of general surgery and provides recommendations for reducing its occurrence.

In the present study, burnout was measured using the MBI-HSS for MP, which is considered the gold standard for measuring burnout [17]. Among attending general surgeons, 69.1% met the criteria for burnout, and the rate of severe burnout was found to be 22%. In addition, nearly two-third of the surgeons had high scores for the EE subscale and about half had high scores for the DP subscale. Since the vast majority of the studies on the burnout of physicians working in the field of surgery examine surgical residents or evaluate all surgical specialties together, it is not sufficient to compare these studies with the present study. Moreover, because of the heterogeneity in classifying burnout across studies, there is no consensus for the interpretation of the MBI-HSS and on how to assess the level of burnout [14, 18]*.*

In a recent meta-analysis evaluating the prevalence of burnout in residents by Low et al. [21], the prevalence of burnout was higher in surgical residents (53.2%) compared with medical residents (50.1%), although the difference was not found to be statistically significant (P = 0.34). In physician subgroup analysis by specialty, it was shown that the highest prevalence rates of burnout were reported in radiology (77.1%), neurology (71.9%), and general surgery (58.3%). Other findings of this study were that older and male residents were significantly more likely to have burnout than their respective counterparts. In a national survey of burnout in 665 general surgery residents, Elmore et al. [1] defined burnout as a high score in either the EE or DP subscale or a low score in the PA subscale and reported a high rate of burnout (69%). They also found that older and women residents, residents working longer hours, and those without a structured mentoring program were more likely to meet the posited criteria for burnout. In contrast, residents who were in their last year of their clinical training and those planning to enter a career in academic medicine were less likely to exhibit burnout. In one of the earliest and largest studies of burnout in surgeons (in which more than half of the participants were ≥ 50 years of age), Shanafelt et al. [6] reported that 39.6% of surgeons were burned out (a high score on either the DP and/or EE subscales) and 30% were identified to positively have symptoms of depression. They found that older age; having more than 50% of professional effort dedicated to nonpatient care tasks, such as education, research, or administration; and having children were less likely to be associated with burnout. However, among surgeons with children, those whose youngest child was aged ≤ 21 were at higher risk for burnout than surgeons having children aged > 21. Other factors independently associated with burnout included working more hours and higher number of nights on call per week, having compensation based on billing rather than a salary, and partner/spouse working as a nonphysician healthcare provider.

To our knowledge, this is the first national study to date to look exclusively at attending general surgeons to assess burnout. In the current study, to avoid confusion associated with the classification of burnout, burnout was assessed both as a continuous variable of each domain and as a dichotomous variable (no burnout vs. burnout). Multivariate analysis revealed that working in a TRH or a state hospital, working ≥ 60 h per week, and not participating in a social activity at least once per week were independent predictors of burnout. The risk factors were almost the same when using a more restrictive definition of burnout. A one-year decrease in age increased the odds of experiencing severe burnout by 4.5%. However, attending general surgeons who worked in a private hospital and had a regular social activity were found to be at lower risk for severe burnout. This result reveals the necessity of improving the working conditions of surgeons practicing in both TRHs and state hospitals and the necessity of organizational, institutional, and national efforts to reduce burnout and promote the quality of care. In addition, considering that the lack of social activities outside of work mostly increases the risk for burnout, individuals need to make necessary efforts to develop the appropriate working patterns to reduce burnout. Developing creative interests and hobbies, allocating time outside of work for family and social activities, and thus finding a balance between personal and professional life are very important [5, 22].

Previous studies have demonstrated that female sex is a risk factor for occupational burnout, especially because of family responsibilities and work-home conflicts [11, 23]. In this study, we reported that female attending general surgeons were more likely to experience burnout than male attending general surgeons; however, gender was not found to be a risk factor. Another significant finding of this study is that workplace was significantly associated with burnout. Attending general surgeons working in a private hospital had lower scores in the EE and DP subscales and were less likely to meet both burnout and severe burnout criteria compared with those working in other hospitals. This may be because of working fewer hours, flexibility in scheduling, having more autonomy, lower patient volume, and higher income [1]. Moreover, many studies have demonstrated that the early years of a professional career are risky periods for burnout [19, 24]. In the present study, specialists were more likely to experience increased EE and DP when compared with assistant or associate professors and full professors. Attending general surgeons early in their career also had the lowest PA. The prevalence of burnout and severe burnout among specialists were 75.1 and 25.7%, respectively. Therefore, it can be concluded that the main factors affecting burnout are the current workload, having less autonomy and flexibility, long working hours, and work-home conflicts, rather than the cumulative exposure to work stressors. Indeed, the results in the present study confirm these predictions. Median EE and DP scores were higher for those who worked ≥ 60 h per week compared with those who worked < 60 h. Moreover, the prevalence of burnout ranged from 64% for attending general surgeons working < 60 h per week to 78% for those working ≥ 60 h per week.

Looked at from another perspective, in a study using representative national samples data from the 6th European Working Conditions Survey, which assessed and quantified the working conditions of employees across thirty-five European countries, Turkey was found to have the highest burnout score [25]. Therefore, it is not surprising that the prevalence of burnout found in our study is higher than the levels reported in many other studies. There may be many reasons for this, but we believe that possible causes of this difference may be economic, sociocultural, and political dynamics at a national level. In addition to the abovementioned stressors and risk factors, Turkish physicians and general surgeons have the added burden of mandatory state service, temporary assignment to hospitals in another city (and therefore frequent relocations and family separation), performance-based payment systems, and threat of workplace violence [26].

Burnout affects not only the mental health and well-being of healthcare providers but also the quality of patient care [1, 10, 19]. As burnout has also been well-known to be associated with major medical errors, it is critical to identify effective and efficient strategies to mitigate burnout as a quality improvement initiative [4, 8]. The high prevalence of burnout and high EE scores among attending general surgeons suggest that burnout is an important psychological outcome and reveals that it requires an organizational, rather than an individual, approach [27]. Since burnout is a problem of whole health care systems, it is difficult to be effectively managed by only stepping in at the individual level. Individually focused solutions have importance to support burned-out physicians but are less likely to have sustainability than strategies that are organizationally centered [28]. Organizations should give priority to working environment improvements for surgeons and the rest of the physician team, such as reducing the clinical workload and/or administrative burdens, streamlining workflows, and fostering quality interactions with patients [29]. Thus, whereas organization-wide strategies make changes in the resources, physician-based interventions focus on individuals such as cognitive-behavioral therapies, stress reduction techniques, training programs for improving communication behaviors. In this study, the key strategies proposed by the participants for reducing burnout and improving professional efficacy are the development a fair salary policy, prevention of violence, reforms for reducing medical malpractice suits, improvement of working conditions, reduction of patient volume, provision of a sufficient number of medical staff, prevention of workplace mobbing, regulation of working hours, communication skills training, and regular psychiatric support.

This study has several potential limitations. Since it relies on self-reported data, the reporting and recall bias inherent to this type of survey methodology are inevitable. Second, it is unknown whether surgeons experiencing burnout are reluctant to complete surveys or are more likely to participate in research, as they believe that cumulative knowledge on this issue is necessary for the solution of burnout. In addition, some busy or elderly surgeons may not want to complete an online questionnaire. Third, because of the cross-sectional design of the study, it is difficult to determine the potential direction of the association and causality. Thus, further longitudinal researches are needed to clarify the relationship between time-related factors and the development of burnout. Fourth, the lack of data regarding some work-related factors (e.g., income, number of nights on call, and subspecialty) and career satisfaction are other limitations. Finally, this study focused only on a single specialty (general surgery) and evaluated the fellows and specialists practicing independently; therefore, the findings may not generalize to other specialties and junior surgeons such as residents.

This study has also several important strengths. It is the largest nationwide study of occupational burnout specifically among attending general surgeons ever conducted. The MBI-HSS is the most commonly used research instrument for assessing burnout. However, there is no consensus on how to evaluate and report the burnout of physicians. To avoid confusion regarding the measurement of burnout, in this study, burnout subscales were assessed as continuous variables, and burnout was also analyzed as a dichotomous variable. In addition, a variety of strategies have also been suggested to promote the well-being of surgeons and to remedy burnout.

## Conclusions

The findings of this national survey study suggest that over two-thirds of attending general surgeons experience burnout and one-fifth are at high risk for severe burnout. Moreover, it is more prevalent, especially in those in their early stages of their careers. Given that this phenomenon is a reversible and preventable condition, efforts to identify at-risk populations and to develop effective strategies to reduce burnout in surgeons are needed. Last, it should not be forgotten that coping with this stress-related condition will not only make a positive impact on physicians but also affect the quality of patient care. Further research is required to identify physician-directed strategies focusing on individuals and structural- or organizational-directed evidence-based interventions targeting the psychosocial work environment. In addition, there is a need to fully examine the impact of these workplace or workflow interventions on surgeon burnout and translate them effectively into clinical practice.

## Supplementary Information

**Additional file 1.** 13-item questionnaire including demographic factors, practice characteristics, leisure activities, and suggestions on how to combat burnout.

## Data Availability

The datasets generated and/or analyzed during the current study are not publicly available but are available from the corresponding author on reasonable request.

## Abbreviations

CI: Confidence interval
DP: Depersonalization
EE: Emotional exhaustion
IQR: Interquartile range
MBI-HSS: Maslach Burnout Inventory-Human Services Survey
MP: Medical personnel
OR: Odds ratio
PA: Personal accomplishment
SD: Standard deviation
TRH: Training and research hospital
