# Supplementary material for: Prevalence and associated factors for burnout among attending general surgeons: a national cross-sectional survey
Source: BMC Health Serv Res. 2021 Jan 7;21:39. doi: 10.1186/s12913-020-06024-5 (PMC7792210; doi:10.1186/s12913-020-06024-5)
Supplement: Supplementary file 1 — Additional file 1. 13-item questionnaire including demographic factors, practice characteristics, leisure activities, and suggestions on how to combat burnout. [file 12913_2020_6024_MOESM1_ESM.docx]

**QUESTIONNAIRE**

**about burnout among attending general surgeons**

1. **Age:** ____________
2. **Sex:**

Female

Male

1. **Marital status:**

Single

Married/Partnered

1. **Children status:**

No

Yes

1. **Academic title:**

Specialist

Asist./Assoc. Professor

Professor

1. **Workplace:**

Training and Research Hospital

State Hospital

University Hospital

Private hospital

1. **Work hours per week:**

<60 hours

≥60 hours

1. **Daily sleep duration:**

<7 hours

≥7 hours

1. **Do you have any comorbidities?**

No

Yes

1. **Smoking status:**

No

Yes

1. **Do you have a specific hobby outside of work?**

No

Yes

1. **Do you participate in any social activities outside of work (at least once a week)?**

No

Yes

1. **Suggestions for reducing occupational burnout:**
   - **………………………………………………………………………….**
   - **………………………………………………………………………….**
   - **………………………………………………………………………….**
   - **………………………………………………………………………….**
   - **………………………………………………………………………….**
